# Food Wastes and Microalgae as Sources of Bioactive Compounds and Pigments in a Modern Biorefinery: A Review

**DOI:** 10.3390/antiox12020328

**Published:** 2023-01-31

**Authors:** Rodrigo Martins, Hélia Sales, Rita Pontes, João Nunes, Isabel Gouveia

**Affiliations:** 1 Association BLC3—Technology and Innovation Campus, Centre Bio R&D Unit, Oliveira do Hospital, 3405-155 Coimbra, Portugal; 2 FibEnTech Research Unit, Faculty of Engineering, University of Beira Interior, 6200-001 Covilhã, Portugal; 3 BLC3 Evolution Lda, Oliveira do Hospital, 3405-155 Coimbra, Portugal

**Keywords:** biomass, bioprocesses, antioxidants, polyphenols

## Abstract

The United Nations 2030 Agenda for Sustainable Development has created more pressure on countries and society at large for the development of alternative solutions for synthetic and fossil fuel derived products, thus mitigating climate change and environmental hazards. Food wastes and microalgae have been studied for decades as potential sources of several compounds that could be employed in various fields of application from pharmaceutical to textile and packaging. Although multiple research efforts have been put towards extracting rich compounds (i.e., phenolic compounds, tocopherols, and tocotrienols) from these sources, they still remain overlooked as two major sources of bioactive compounds and pigments, mainly due to inefficient extraction processes. Hence, there is a growing need for the development of optimized extraction methods while employing non-organic solvent options following the main principles of green chemistry. This review will focus on delivering a clear and deep analysis on the existing procedures for obtaining bioactive compounds and pigments from food wastes derived from the most consumed and produced fruit crops in the world such as apples, oranges, cherries, almonds, and mangoes, and microalgal biomass, while giving light to the existing drawbacks in need to be solved in order to take full advantage of the rich properties present in these two major biorefinery sources.

## 1. Introduction

In modern society, the motivation for development of alternative solutions for fossil fuels and synthetic materials is driven by several key factors: (1) the diminishing reserves of recoverable oil; (2) the increasing of demand and prices of petroleum-derived fuel; (3) the concerns over increasing greenhouse gas emissions that affects the global climate; (4) the growing food needs, and (5) the desire for global energy independence and security [1]. In addition, the goals set on the 2030 Agenda for Sustainable Development signed by all the United Nations (UN) member states have created a driving force for change [2]. To solve such issues, biorefineries could play a vital role. A biorefinery is a facility that integrates biomass conversion processes for sustainable processing of biomass into value-added bio-based products (food, feed, chemicals, and materials) while taking full advantage of the various components in biomass (such as food wastes and microalgae) and their intermediates, maximizing the value derived from the biomass feedstock [1]. Most importantly, in contrast to the petroleum-based products, biorefinery products are non-toxic, biodegradable, reusable and recyclable. Therefore, in this review article two major contributors to biorefinery production (food wastes from major crops produced and consumed in the world, and microalgae) are analyzed, including an up-to-date overview of extraction methods used, current applications, market analysis and future prospects.

Each year, around 45% of total fruit and vegetable production is lost during various stages of the food chain: field (infestation, post-harvest), processing (handling, distribution, and retail), and consumption (consumer levels). It is estimated that household operations produce up to 42% of food waste, 39% is due to the food industry, 14% is lost in the food service sector (prepared to consume food, restaurants, and hotels), while 5% is wasted during production [3]. In addition, it is estimated that US $1 trillion capital loss is attributed to this food wastage. Therefore, adaptation of circular bioeconomy for reduction of food waste would be of great significance and this can be integrated at any stage of waste generation [4].

As aforementioned, the food industry as a whole is one of the main contributors for food losses and waste generation [3]. As per a study conducted by Nayak et al. [5], it is estimated that approximately 26% of food wastes are generated from the drinks industry (making it the biggest contributor to food waste generation), 21% is generated by the dairy industry, 14.8% from fruit and vegetable production, 12.9% from the manufacturing and processing of cereal, 8% from meat product processing and preservation, 3.9% from the manufacturing and processing of vegetable and animal oils, 0.4% from fish product processing and preservation, and others (12.7%) [5]. Despite their origin, such wastes are usually characterized by high moisture content, high biological instability and high organic loading, which promotes microbial activity and makes these wastes difficult to handle [5].

To deal with such wastes, during past decades, research and efforts have been made for the development and valorization of bioactive compounds present in food wastes such as leaves, peels, seeds, and pulp, thus minimizing the environmental impact and promoting the recovery of rich compounds that are potentially marketable [6]. Hence, while the use of several fruits and vegetables in several industries can generate high volumes agri-food wastes, such agri-food wastes may contain different types of bioactive compounds with marketable applications [7] that can be extracted through various extraction techniques (conventional or non-conventional) [8]. The valorization of such wastes has been one of the main focus of the food industry, which has focused much of its energy on the development of healthier foods using bioactive compounds—substances that have biological activity related to their ability to modulate one or more metabolic processes that promote better health conditions—thus decreasing diseases related to the consumption of low nutritional and high caloric foods [6].

Alongside food wastes, microalgae have been recognized as a very important group of photosynthetic microorganisms for their many attributes and roles in nature, including as biomass for biorefineries. Microalgae are autotrophic microorganisms that consume CO_2_, light, and inorganic nutrients to produce biomass, rich in primary metabolites such as lipids, carbohydrates, proteins, and pigments. Microalgae can produce high-value compounds such as polysaccharides, poly-unsaturated fatty acids (PUFAs), carotenoids (lutein, zeaxanthin, and astaxanthin), and vitamins with very high nutraceutical and pharmaceutical potentials [9]. Hence, it has been demonstrated that microalgae have a large potential in biotechnology as a source of various macromolecules (proteins, carbohydrates, and lipids) and value products (pigments and PUFAs) [10].

Several algae-based compounds, such as polysaccharides, steroids, fatty acids, carotenoids, halogenated compounds, and peptides, have a large number of biological properties including anticancer and antimicrobial activity [11]. These bioactive molecules bind to diverse sites, suppressing cell division, or inducing apoptosis via activation of specific cellular pathways, which are important for the inhibition of different diseases such as cancer [9]. For instance, natural astaxanthin, a bioactive pigment that can be found in microalgae species [12], has several health benefits and is considered a “super antioxidant” [13]. Reviews suggest that natural astaxanthin is a superior antioxidant that is able to reduce inflammation, improve gastrointestinal health and the immune-system, potentially act against atherosclerotic cardiovascular disease, reduce the chance of heart attacks, Alzheimer’s disease, and other neurological diseases, inhibit the growth of cancer cells and tumors, protect from UV-radiations, and decrease the chance of idiopathic infertility [13].

Although microalgae exhibit such valuable properties, one of the main concerns is the scale-up of processes to take full advantage of the valuable compounds that microalgae possess [10]. Hence, research on sustainable processes that can take advantage of all the macromolecules in a circular and “green” process is highly needed.

## 2. Methodology

This research work seeks to give a better understanding about the current state of existing methods and the solvents used to extract valuable bioactive compounds and pigments from microalgae and food waste sources. 

To conduct this review, the databases chosen were ScienceDirect and Web of Science. In addition, to add valuable information to the review, documents of the Food and Agriculture Organization (FAO), UN, BASF, and Hoffman-La Roche were analyzed. The selected articles for the redaction of this review were based on a timeframe between 2010 and 2023. In Table 1, a summary of keywords/search terms used in the research of literature, and the results obtained for the ScienceDirect and Web of Science databases is presented. For the given search results, inclusion and exclusion criteria were employed, such as reading the title, abstract and keywords to allow the authors to select articles compatible with the main research theme.

## 3. Innovative Extraction Methods

The extraction process of bioactive compounds and pigments is one of the most important factors in the production of new products, since they have an impact (positive or negative) on the environment. Conventional extraction techniques such as maceration, Soxhlet, solid– liquid extraction (SLE) or liquid–liquid extraction (LLE), are characterized by high volumes of toxic solvents and long extraction times [14,15]. Furthermore, these techniques often have low extraction yields and low selectivity of the desirable bioactive components. In addition, due to the use of toxic solvents, these processes are considered very unfriendly to the environment. To overcome the limitations of these conventional extraction methods, non-conventional techniques such as microwave-assisted extraction (MAE), ultrasound-assisted extraction (UAE), high-pressure extraction (HPE), pulsed electric fields (PEF), and supercritical fluid extraction (SC) have been studied for the extraction of rich bioactive compounds from food wastes [14] and microalgae [16].

In Table 2, a summary of the main advantages and disadvantages associated with extraction techniques often used in the extraction of valuable compounds from biomass (i.e., food wastes and microalgae) is presented [17].

As shown in Table 2, whilst conventional extraction techniques may present higher extraction times and rely on the use of high volumes of solvent, non-conventional techniques often require specialized equipment—associated with high costs and initial investment—and may face scalability issues [18].

Another important factor to take into account concerning an extraction process is the solvent used in the biomass matrix. To make an assertive solvent choice, solvent selection guidelines (SSGs) have been reported classifying the solvents as useable, preferred, substitution advisable, hazardous, undesirable, and banned [19]. Furthermore, scientists have created a criteria-based algorithm where the principles of green analytical chemistry are taken into account. From the 151 most used solvents evaluated, polar solvents such as ethanol, 1-propanol, acetone, acetonitrile, 2-propanol, and methanol are listed as environmentally safe, making them top of the list of green chemicals, while nonpolar chemicals including pentane, dichloromethane, hexane, benzene, carbon tetrachloride, and chloroform are considered in the highly hazardous, undesirable, and banned chemicals groups. Additionally, xylene, heptane, toluene, and chlorobenzene solvents are considered usable but also very problematic [19].

In the extraction of bioactive compounds from natural matrices, aqueous-organic solvents such as hexane, benzene, methanol, chloroform, petroleum ether, and acetone are often used. Usually, the use of these conventional solvents is related to multiple issues such as toxicity, volatility, and flammability [20]. Therefore, they can be harmful to the environment, operator and consumer health. Hence, researchers are keen to develop new solvent alternatives to help improve the overall process sustainability. Ionic liquids (ILs), deep eutectic solvents (DES), and more recently natural deep eutectic solvents (NADES) have played a major role.

ILs can be defined as salts formed with an organic cation and an organic or inorganic anion, with melting points lower than their initial constituents, usually close to 100 °C. Moreover, ILs present desirable thermodynamic properties such as low vapor pressure, thermal stability, adjustable viscosity, miscibility, solubility, and extraction capacity for many organic and inorganic compounds. However, there are difficulties in introducing them in agro-industries because they are not regulated by FDA (Food and Drug Administration, White Oak, MD, USA) or European legislation, and their effects have not been totally elucidated [20].

On the other hand, DES can be considered an alternative to IL since they present similar thermodynamic properties, are easily synthesized, are less harmful to the environment, and present less toxicity. These solvents are formed by complexing a hydrogen bond acceptor (HBA), such as quaternary ammonium, with a hydrogen bond donator (HBD), such as urea, carboxylic acids or ammine. Despite their properties, DES selection is wise. As studied by Morais et al. [21], the cholinium-based DES, cholinium chloride, as an HBA, and organic acids, namely acetic, citric, lactic, and glycolic acids as HBDs, were evaluated, and it was concluded that theses cholinium-based DES showed an intermediate toxicity and could be considered as “moderately toxic” solvents. Their toxicity is clearly related to the concentration of the organic acid. In addition, they were also shown to be more toxic than their corresponding ILs, namely cholinium glycolate, cholinium acetate, cholinium dihydrogencitrate, and cholinium lactate. Thus, the right selection of DES is still very significant to the overall safety of the extraction process.

When natural components are used for DES synthesis, usually plant primary metabolites (e.g., sugars), they are called natural deep eutectic solvents (NADES). Compared to the previously discussed solvents, NADES have the advantage of emulating the natural solvation method for water-insoluble primary and secondary metabolites in plants. Therefore, NADES act as an alternative liquid phase in nature [20]. Furthermore, NADES have a lower melting temperature than the starting material and some of them form transparent liquids at ambient temperature, which make these solvents easy to prepare and use. High viscous NADES solutions, which can impede extraction rate and time, can be mitigated by high temperatures and/or a small proportion of co-solvent, such as water [22]. Moreover, since NADES are synthesized by natural components, which are inexpensive, abundant, and recyclable biomaterials, they are seen as non-toxic solvents, making them highly compatible with food, pharmaceutical, and cosmetic formulations [20]. To emphasize the non-toxic nature of NADES, some studies have reported that no cytotoxicity has been detected [23]. Additionally, reports also show that NADES are biodegradable [24]. Therefore, NADES are seen as less toxic and more biodegradable than the other two new generation solvents discussed (ILs and DES) [23], making them a great and suitable solvent choice for the extraction of bioactive compounds and pigments from biomass sources (i.e., food wastes and microalgae).

## 4. Bioactive Compounds

### 4.1. Bioactive Compounds from Food Wastes

Food wastes are one of the main sources of bioactive compounds in the world since through intensive research it has become clear that production waste such as peels, seeds, and pomace, contain high-value bioactive compounds [25]. This approach represents the main principles of a circular economy in any agri-food industry since it reduces pollution levels and increases the competitiveness of agri-food industries, where a wide variety of products can be obtained using waste as a raw material. Hence, in addition to the direct ecological benefits, there is also an economic gain for companies, linking integration of recovered ingredients into human food supply chains [25].

Since the fruit and vegetable industry is one of the main contributors for food losses and waste generation [3], we present a much needed in depth review of innovative techniques used to extract bioactive compounds from different food wastes generated in the consumption and processing of the most consumed and produced fruit crops in the world.

#### 4.1.1. Bioactive Compounds from Apples

Produced in large quantities each year, apples (*Malus domestica*) are considered an important source of bioactive compounds [15]. Apples are usually processed for juice recovery leading to large quantities of by-products (30% of raw material) known as apple pomace (fruit pulp, peels, and seeds) [26].

As per Carson et al. [27], apple pomace usually consists of 2.2–3.3% seeds, 0.4–0.9% stems, 70.0–75.7% apple flesh and 20.1–26.4% rice hull (added before pressing juice as a processing aid). Although considered a by-product, apple pomace still contains different functional components such as polyphenols (or phenolic compounds), polysaccharides, vitamins (i.e., vitamin E), fibers, and triterpenes [26]. These compounds have been researched over the last decades for their great health-promoting roles such as antioxidant, anti-histaminic, anti-tumor, and anti-cancer properties [15]. Therefore, the extraction of rich compounds from apple pomace is a crucial process for its valorization, and should be an environmentally friendly process that not only creates value through the extraction of marketable high-value compounds but also prevents environmental hazards [6,14].

In obtaining extracts and bioactive compounds from apple wastes, the extraction method is a relevant factor since this process insures the adequate separation of the bioactive compounds from the cellular matrix. The efficiency and the selectivity of the extraction process depends largely on the technique, the solvent, the energy input, and the agitation that can improve the chemical solubility and efficiency of mass transfer [26]. The traditional techniques (maceration or Soxhlet extraction) are widely used because they are relatively cheap and easy to operate, but they usually require long extraction times, high energy consumption, intensive manual procedures, and may also have low efficiency [26]. Thus, it is necessary to develop innovative and scalable methods leading to implementation in various industries. Methods such as pressurized liquid extraction (PLE), UAE or MAE have been introduced and studied in the extraction of bio compounds from food wastes, particularly from apple pomace.

Cristina-Gabriela et al. [26], evaluated different extraction techniques such as PLE, UAE, and MAE in the pomace of four apple varieties (i.e., Gala, Golden, Granny Smith, and Pink Lady) and the extracts obtained were characterized by liquid chromatography. The results obtained showed that MAE had the highest extraction yield. Moreover, in a different study conducted by Rezaei et al. [15], MAE was faster than the conventional methods (maceration and Soxhlet) when used in the extraction of polyphenolic compounds from apple pomace.

Although the MAE method has its advantages, there have been some reported drawbacks [18], such as the handling and processing of limited sample volumes, that can possibly make difficult the viable scale-up and implementation of this process for processing bulk quantities [18]. Furthermore, it has been reported that uniform heating is rarely achievable in conventional microwave systems, often giving rise to both unprocessed and severely overheated spots [14]. Moreover, different parameters such as the extractant nature, microwave irradiation power, extraction temperature and time, and the solvent-to-feed ratio, must be optimized and considered for an efficient MAE extraction of bioactive compounds from different cellular matrices [18].

In Table 3, a summary of different studies conducted for the extraction of bioactive compounds from wastes of different apple varieties is presented.

From Table 3, it can be seen that the conventional techniques usually require longer extraction times, which usually lead to higher energy consumption, and use conventional solvents [35]. Some of these drawbacks can be overcome by non-conventional techniques such as UAE and MAE methods which use lower solvent quantities and require lower extraction times. Despite their advantages, these extraction methods are mostly done at a laboratory scale, and in several studies the use of organic solvents is still employed [25,27].

Despite the interesting studies conducted on the use of these green and innovative extraction methods in several food wastes (including apple wastes), few studies have used non-organic solvents (ILs, DES and NADES) in these extraction methods, and even fewer scale-up studies have been reported [35]. This is an important factor to keep in mind since it increases the interest of industries in the hidden potential of food wastes. Furthermore, in developing or under-developed countries, where malnutrition is at all-time high, such reasonable and viable nutrition sources are important and should be given proper attention [36]. Moreover, the valorization of food residues in general could solve several issues that these countries face with respect to the proper treatment of organic food wastes [37].

As shown in Table 3, several bioactive compounds extracted from apple wastes are reported in the literature such as phenolic compounds (or polyphenols) and tocopherols (or vitamin E). The phenolic compounds can be sectioned into five major classes, namely, hydroxycinnamic acids (primarily chlorogenic acid), flavanols (catechin, epicatechin, and anthocyanidins), flavonols (mainly different quercetin glycosides), dihydrochalcones (mainly phloridzin), and anthocyanins (a reddish bioactive pigment present in the skin of some red-flesh apple varieties [38]) [15].

Phenolic compounds (such as those present in apple pomace) are hydrophilic bioactive compounds [39] that have the capacity to inhibit Reactive Oxygen Species (ROS) (for instance interrupting the cascade of free radical reactions in lipid peroxidation), in addition to other antioxidant effects, depending on their structure [40]. Such capacity represents a great advantage for the inclusion of phenolic compounds in dermal products, since using antioxidants in this type of product is required to maintain their quality throughout their shelf-life [40]. Moreover, phenolic compounds found in apple pomace have also been reported for anti-inflammatory, anti-proliferative, anti-tumor, and cardioprotective properties.

For instance, phloridzin is a polyphenol that has been proven as a competitive inhibitor of intestinal glucose and also as an anti-diabetic compound [41]. In addition, phloridzin has antioxidant capabilities that could play an interesting role in pharmaceutical and food applications [42]. Moreover, phenolic acids (e.g., gallic acid and caffeic acid) present in apple pomace, have also shown potential as antioxidants mainly through radical scavenging via hydrogen atom donation [43].

On the other hand, tocopherols (vitamin E), which are lipophilic bioactive compounds [39], are also among the most important natural antioxidants found in apple, and more specifically in apple seeds. From a study conducted by Akšic et al. [30], several apple varieties were studied in order to determine their tocopherol and fatty acid content. Results showed that the maximum value for vitamin E found in one apple variety was 1.811 μg/g of dry weight. This compound could play a major role in human health since it can impact the human neurological system and also prevent heart disease and prostate cancer.

#### 4.1.2. Bioactive Compounds from Citrus

Citrus fruits are among the most extensively cultivated fruits in the world. Orange (*Citrus sinensis*), mandarin (*Citrus reticulate*), lemon (*Citrus limon*), lime (*Citrus aurantiifolia*), and grapefruit (*Citrus paradisi*) are some of the important commercially grown citrus fruits, mostly due to their numerous phytochemicals and bioactive components, which have been studied for their health-promoting properties [44].

Citrus fruits have acquired great interest because of their high content of bioactive compounds such as AsA (ascorbic acid or vitamin C) and polyphenols, mainly flavonoids. Flavanones (phenolic compounds) such as naringin and hesperidin are usually found in tissues and peels of citrus fruits, displaying numerous therapeutic advantages due to their antioxidant, anti-inflammatory, and anti-carcinogenic properties [44].

Orange (*Citrus sinensis*) is one of the most cultivated citrus fruits in the world with an estimated production of 72 million tons in 2014 [45]. Like most of the fruits covered in this review, a large quantity of oranges (almost 50%) is used for juice processing [45]. The disposal of orange fruit segments without proper treatment is dangerous to the surroundings because of their undesirable and unhygienic nature. Since citrus juice (including orange juice) production leads to the generation of waste—including peels (50–55% of the total fruit mass), seeds (20–40% of total fruit mass), pomace, and wastewater (portions of spoiled fruit, seeds, pulp, and peels)—which is practically 50% of the fresh fruit mass, the development of innovative ways to treat these wastes is ever more relevant [45].

In Table 4, studies focused on the extraction of valuable bioactive compounds from orange wastes (i.e., phenolic compounds, polyphenols, and tocopherols) found in the literature are summarized.

Athanasiadis et al. [46], studied the parameters that affect the extraction of bioactive compounds such as polyphenols, AsA, and hesperidin from orange peel. In this study, antioxidant properties were optimized using a response surface methodology (RSM), the main variables being the extraction temperature, time, and composition of the extraction solvent. In addition, the effects of two more techniques (UAE and PEF) were examined separately and combined to determine whether they can enhance the extraction of the desired compounds. Results showed that orange peels are an excellent source of many bioactive compounds, since the extracts contained hesperidin (1.626% of dry weight) for 180 min of PEF extraction at 65 °C, polyphenols (3.471% of dry weight) for 30 min of PEF combined with UAE extraction at 35 °C, and AsA (1.229% of dry weight) for 180 min of UAE extraction for 80 min.

Montero-Calderon et al. [47], studied the use of UAE to extract bioactive compounds from orange peel. The results demonstrated that the optimal conditions for the UAE of bioactive orange peel compounds were 400 W, a time of 30 min, and 50% ethanol in water. In these conditions, it was possible to obtain a vitamin C concentration of 53.78 mg/100 g, and a phenolic concentration of 105.96 mg/100 g, which were lower than the results reported by Athanasiadis et al. [46].

In summary, orange peels contain a plentitude of bioactive constituents, such as polyphenols (hesperidin, naringin, nobiletin, and tangeretin) and flavoring compounds, that have been shown to have great antioxidant properties. However, as shown in Table 4, studies employing other non-organic solvent options are still an untapped research theme.

On the other hand, orange seeds, which are a rich source of protein, are another by-product of orange processing and constitute of approximately 4.8% of dried citrus pulp [50]. Citrus seeds contain bioactive phytochemicals such as polyphenols, flavonoids, antioxidants, limonoids [49], and tocopherols [48]. Owing to the presence of these functional compounds, citrus seeds have many applications in food, pharmaceuticals, and cosmetics [35].

#### 4.1.3. Bioactive Compounds from Cherries

*Prunus avium* L., known as sweet cherry, is a native from the area between the Black and Caspian seas of Asia. According to the FAO, Turkey is the world’s biggest producer of cherries (480,748 tons), followed by the United States of America (384,646 tons), Iran (200,000 tons), and Italy (104,766 tons), while the global sweet cherry production is around 4.0 million tons per year [25].

Sweet cherries are composed of an edible and thin protective red, maroon, or purplish-black skin (exocarp), an edible red and sometimes white succulent flesh (mesocarp) and an inedible seed (endocarp) [45,46]. Most of sweet cherries are produced to be consumed as fresh fruits, but since they are seasonal fruits, they are not available year-round in the supermarket, and are usually frozen, brined, canned, dried, and processed into jams or juices [51]. Like the previous cases analyzed in this review, the processing of cherries produces by-products.

For instance, cherry seeds that result from processing sweet cherry into sweets, juices, and jams, are one of the major cherry processing by-products [25]. In fact, cherry seeds constitute more than 60% of the fruit weight [52]. Generally, seeds are considered production waste, and have gained strong interest due to the environmental aspects related to disposal.

On the other hand, the main by-product in cherry processing is cherry pomace, which consists of skin and flesh obtained after juice pressing, and makes up to 15–28% of the initial fruit [53].

In Table 5, a summary of different studies found in the literature focusing on the extraction and valorization of cherry wastes is presented.

Sweet cherry seeds were the focus of a study conducted by Dulvanska et al. [25], where the extraction of polyphenols was evaluated. The extraction process was investigated by employing different conditions to optimize extraction parameters, such as solvent and temperature, for the maximum yield of extracted bioactive compounds. The results showed that the best extraction technique for phenolic compounds was an aqueous ethanol solution at 80 °C. In addition, it was reported that temperatures above 80 °C were not beneficial, since they can degrade some families of phenolic compounds, reduce the extraction of total phenolic compounds, and consequently the potential of the overall extraction. On the other hand, it was concluded that temperatures of 80 °C also favor the recovery of flavonoids.

Besides phenolic compounds, studies [54,55] have been done focusing on the extraction of tocochromanols from cherry seeds, which have shed light on two different extraction methods (Soxhlet and UAE). Both studies were able to extract bioactive compounds with antioxidant capabilities. While both studies employed organic solvents, they were able to introduce valorization of the seeds of different cherry cultivars. In the study done by Górnas et al. [55], the influence of the cherry specie in the results obtained for the extraction of bioactive compounds was evaluated. It was found that the cherry species appeared to be directly associated with tocopherol profile.

Concerning another by-product of cherry processing, cherry pomace, multiple studies have been done. For instance, in a study conducted by Gonçalves [50], data revealed that sweet cherry extracts obtained from Hedelfinger, Saco and Maring cherry varieties, were more effective in the inhibition of α-glucosidase activity than acarbose, one of the most well-known drugs commercialized as an enzyme inhibitor for type two diabetes. Furthermore, from the results obtained, it was concluded that sweet cherry extracts have great biological potential, mainly due to their antioxidant activity against free radical species and protecting cells against oxidative damages, and may even be used as a therapeutic in the treatment of inflammatory diseases (such as diabetes, gout, and arthritis), hemolytic anemia, cancer, neurological and cardiovascular pathologies [50].

Šaponjac et al. [56], conducted a study in which bioactive compounds extracted from cherry pomace were encapsulated in whey (WE) and soy (SE) proteins and incorporated in cookies, replacing 10% (WE10 and SE10), and 15% (WE15 and SE15) of flour. Total polyphenols, antioxidant activity and color characteristics of enriched cookies were followed during 4 months of storage, and it was concluded that total polyphenols of WE10, SE10, WE15 and SE15 showed a slight increase, and antioxidant activity decreased. Overall, encapsulated sour cherry pomace positively influences the functional characteristics of fortified cookies and their preservation [56].

Nowadays, there are more attempts to use environmentally friendly extraction methods, using more sustainable approaches, resulting in the maximum recovery of bioactive compounds, using ecological solvents, and reducing processing costs. From the studies found, hydrophilic and lipophilic bioactive compounds such as phenolic compounds and tocochromanols, were present in several cherry by-products. While the extraction of bioactive compounds from cherry wastes using green solvents is still very much undeveloped, this fruit shows great potential for bioactive compound extraction and application in cosmetics and pharmaceuticals offering health benefits [54].

#### 4.1.4. Bioactive Compounds from Almond

Almonds (*Prunus dulcis (Mill.), D.A. Webb or Amygdalus communis L.*) are largely used in the preparation of several traditional bakery and confectionery products, including almond cookies, marzipan, and almond milk [58]. The almond fruit is the most produced nut worldwide, due to its exceptional nutritional value (low sugar content, high levels of proteins, unsaturated fatty acids, vitamins, and minerals, as well as health-enhancing phytochemicals).

Besides the fruit, almond production involves the generation of several by-products that are normally discarded, accounting for 0.8–1.7 million tons of shells and more than 6 million tons of almond hulls [59]. Recent studies show that *Prunus* species may protect against metabolic syndrome, which includes sensitivity to insulin, visceral obesity, dysregulated metabolism of glucose and lipid, and hypertension. They can also be used to treat stress, immune problems, and anemia, as well as improve brain function.

The first processing step of the almond fruit is the removal of the brown skin by means of blanching in hot water and subsequent mechanical peeling. The almond skin accounts for 6–8% of the seed, and its main use is for cattle feed. The blanching water represents a processing waste, and the producers must incur costs for its disposal [58]. Consequently, the accumulation of almond by-products is causing an increasing concern about their processing, and novel solutions are required to add value to these residues, with the aim of improving the economic profit and environmental sustainability of the process [59].

Various studies have shown that almond by-products (kernel, skin, and shell) contain bioactive compounds such as phenolic compounds (flavonoids and phenolic acids) and terpenoids (sterols and triterpenoids), whose composition and quantity depend on factors such as geographical distribution, origin, environmental conditions, exposure to pests, UV radiation, harvest maturity and the extraction process. These by-products are a source of potent antioxidants for the control of oxidative processes [59].

For instance, reports have shown the total content of phenolic compounds in the kernel (8 ± 1 mg QCE/g ethanolic extract to 8.1 ± 1.75 mg CE/g ethanolic extract), skin (87.8 ± 1.75 mg CE/g ethanolic extract to 88 ± 2 mg QCE/g ethanolic extract), shell (38 ± 3.30 mg GAE/g methanolic extract) and hull (71.1 ± 1.74 mg CE/g ethanolic extract to 78.2 ± 3.41 mg GAE/g methanolic extract) of the almond (*Prunus amygdalus L*.). Furthermore, the almond of *Prunus serotina* has been studied as a source of lipids, raw fiber, and carbohydrates, in addition to containing vitamin E and minerals such as Ca, Fe, Mg, P, K, Zn, and Na. Moreover, the *Capulin* almond stands out for its high level of α-eleostearic acid (27%), which is effective in the suppression of the growth of cancer cells, and possesses antihypertensive properties due to the presence of vasodilator compounds such as ursolic acid and uvol [60].

In Table 6, different extraction procedures applied to almond by-products (almond hull, almond kernel, almond shell, and almond skin) for recovery of bioactive compounds are summarized [60].

#### 4.1.5. Bioactive Compounds from Mango

*Mangifera indica L.*, commonly known as mango, is one of the most consumed fresh fruits in the world, with production occurring in more than one hundred countries. In fact, mango is the second most produced tropical fruit in the world, and India is its largest producer (accounting for around 45% or 15 million tons), cultivating more than 1000 varieties of the tropical fruit [61].

The year-round availability of mangoes is attributed to several factors, including the diverse climatic conditions in which the fruit can be grown. The demand for mangoes is also on the rise due to the higher percentage of health-conscious consumers [62]. Mango chemical composition varies according to culture, selection, maturation stage conditions, and other factors, but it is generally mostly composed of water, carbohydrates, organic acids, minerals, proteins, vitamins (AsA), carotenoids, and other pigments [61]. In addition, with the increasing interest in the characteristics of mangoes (flavor, health benefits), it is ever more important to study different bioactive compounds present in mangoes and mango food wastes (peel, leaves, and kernel) [62].

In Table 7, a summary of studies conducted for mango waste valorization, through several extraction methods, is shown.

In the study conducted by Sanchez-Sanchez et al. [63], extracts obtained from mango leaves were successfully impregnated into textile polyester using supercritical carbon dioxide. In this study, used leaves of *Mangifera indica L.* were submitted to enhance solvent extraction using a mixture of CO_2_/methanol (50%) at 120 bar and 100 °C, obtaining mango leaf extracts (MLE). MLE were then used in conjunction with supercritical CO_2_ to impregnate a polyester textile, and results showed that the extracts presented antioxidant, bacteriostatic, and bactericidal activities [63]. Mangiferin is also one of the predominant polyphenols in mango leaves and has multiple pharmaceutical properties, such as antioxidant, antibacterial, antifungal, antidiabetic, immunomodulatory, anti-inflammatory, and analgesic properties, with potential uses in the treatment or prevention of chronic diseases including cancer, neurodegenerative, and cardiovascular diseases [65].

Other valuable phenolic compounds with interesting pharmaceutical properties, such as quercetin, gallic acid, gallotannins, and iriflophenones, have been identified in mango leaves. The high content of potent antioxidant polyphenols is the reason for the great potential of mango leaf extracts in cosmetic, nutraceutical, pharmaceutical or food applications [63].

Mango peel is another major by-product of the mango processing industry, and accounts for approximately 15–20% of the total weight of fresh fruit [65]. This mango by-product has been studied for the presence of bioactive compounds. In a study conducted by Ajila et al. [65], peels from Raspuri and Badami mango varieties were prepared with 80% acetone to obtain mango peel extracts. Results showed that the acetone extracts of mango peel contained polyphenols. Furthermore, the extracts obtained from different peel varieties showed differences in antioxidant activities that may be due to variations in the peel composition; more precisely in the content of antioxidants like polyphenols [65].

In a study conducted by Coelho et al. [66], different methods of maceration were evaluated in the production of mango peel liqueurs from two different mango varieties (Haden and Tommy Atkins). The two maceration techniques used were alcoholic maceration and maceration with pectinase. Results showed that alcoholic maceration in wine ethanol (65% *v*/*v*) produced liqueurs with higher phytochemical and antioxidant content, while maceration with pectinase resulted in liqueurs with higher quercetin-3-O-glucopyranoside content. In relation to mango varieties, Haden liqueurs presented higher bioactive content than Tommy Atkins liqueurs. The main bioactive compounds found were flavanols (epicatechin-gallate and epigallocatechin-gallate), flavonols (quercetin-3-O-glucopyranoside and rutin), and phenolic acids (gallic acid, o-coumaric acid, and syringic acid). Thus, the production of liqueur enabled the recovery of an important part of the bioactive content of mango peels, suggesting an alternative for the recovery of antioxidant substances from this by-product [66].

In an interesting study done by Manhongo et al. [67], an economic assessment of mango process feasibility, economic viability, and environmental impacts of model integrated biorefineries for co-producing bioenergy and bioactive compounds were evaluated using three scenarios for integration with dried mango chips [67]. The study concluded that in both techno-economic and environmental life cycle assessment, mango waste is an attractive bioresource for co-producing pectin and polyphenols, as well as bioenergy, in a self-sufficient manner. Although demonstrating the lowest environmental impacts and the least capital investment, mango processing for production of bioenergy was the least attractive option in terms of profitability. In contrast, the co-production of polyphenols with heat and electricity was shown to be the most capital-intensive option and presented the highest environmental impacts, yet the scenario is the most favorable in terms of profitability, demonstrating the value of bioactive compound production from mango fruit waste [67].

### 4.2. Bioactive Compounds from Microalgae

Microalgae are photosynthetic organisms that include more than 7000 species that grow in a wide variety of habitats. They can develop in such diverse environmental conditions such as freshwater, brackish water, and sea water [13]. Hence, microalgae have been introduced as a source of biomass incorporated in third-generation biorefineries. Microalgae are recognized as rich raw materials since they are composed of a plethora of bioactive compounds, namely pigments (such as carotenoids, chlorophylls, and phycocyanin), proteins, polysaccharides, and long fatty acids, which have been applied over the years in different industries, including cosmetics, human food, and energy [68]. Due to their unique properties, microalgae could play an important role worldwide in the production of high-value-added products, foods, feeds, fertilizers, polymers, and biofuels, in addition to having some other environmental assets such as wastewater bioremediation and carbon dioxide mitigation. The microalgae-based bioeconomy should include environmental benefits with the co-production of high-value-added products to offset the high production costs of microalgae cultivation and processing [69].

Although land plants are highly exploited for their production of natural products, the diversity of compounds produced by algal species is estimated to be over 10 times greater than those produced by land plants [70]. Furthermore, unlike terrestrial plants, microalgae lack highly resistant cell wall components with no stem or roots, allowing easier degradation and, consequently, easy exploitation of valuable bioactive compounds [66]. Moreover, microalgae production is not affected by the climatologic season, leading to higher yields of production when compared to land plants [71]. For these reasons, algal-derived natural products (specifically including bioactive compounds) are a great untapped resource for multifaceted uses spanning health, and other industrial sectors [70].

Microalgae are also known worldwide for their application as bioremediation players due to their outstanding capacity to sequester carbon dioxide (CO_2_) from the atmosphere, and as raw materials considering the large variety of bioactive compounds in their constitution [68], particularly as feedstock to produce oils. The oil obtained from microalgae can be used directly as fuel or be chemically transesterefied into biodiesel [72]. In addition, other biofuels, such as ethanol and methane, can be produced through fermentation and anaerobic digestion, respectively.

Algal oil can be used in different sectors in addition to the production of biofuels, for example, in the nutraceutical sector as nutritional supplements and in several cosmetics products. The considerable lipid content in microalgae allows the production of alternative renewable cleaner fuels [73].

In Table 8, a summary of studies focusing on the extraction of bioactive compounds from several microalgae species is shown.

Lu et al. [16], used *Chlorella* sp. pretreated with three different aqueous deep eutectic solvents (aDESs): aqueous choline chloride-oxalic acid (aCh-O), aqueous choline chloride-ethylene glycol (aCh-EG), and aqueous urea-acetamide (aU-A), to study the effect of aDESs pretreatment of microalgae biomass in terms of lipid recovery rate, total carbohydrate content, fatty acid composition, and the thermal chemical behavior of biomass. Results indicated that lipid recovery rate was increased from 52.03% of untreated biomass to 80.90, 66.92, and 75.26% of the biomass treated by aCh-O, aCh-EG and aU-A, respectively. However, there were no major changes observed in fatty acid profiles of both untreated and treated biomass, specifically palmitic acid, palmitoleic acid, and stearic acid under various pretreatments [16].

Young-Hoo et al. [74] showed that the lipid extraction effect of [Bmim][MeSO4] from *Chlorella vulgaris* combined with UAE pre-treatment exhibited two-fold and 1.6-fold higher lipid extraction than with a classic approach (Soxhlet).

The complete fractionation and valorization of all microalgae components should be accomplished to take the maximum advantage of the algal biomass. Such a task could be accomplished by optimization of extraction techniques and by market size evaluation. While microalgae-derived biofuels are attracting ever-increasing interest, many studies are showing them to be unviable economically if not followed by the valorization of the microalgae value-added compounds with far more interesting applications, mostly in the pharmaceutical and cosmetic sectors, but also as fine chemicals [71].

## 5. Bioactive Pigments

Color affects various aspects of human life from the clothes we wear to the process of photosynthesis in plants [77]. Color vision for humans depends on molecules called pigments because of their ability to absorb UV radiation with wavelengths in the range of 380 to 750 nm [78]. Thus, life on earth is dependent on the existence of pigments, and in particular biological pigments [77].

In 2018, the global demand for pigments was approximately 9.7 million tons [79], and in 2014, 45% of the total demand was linked to the production of paints, the food sector, and the pharmaceutical sector. Healthier lifestyles, and the growing market for natural food colorants, propelled by an increased awareness on the harmful influence of synthetic colorants and the resultant boost in the demand for natural colorants, and restrictions and limitations in manufacturing and trading of synthetic colorants, which drives toward alternate natural colorants, an increase in demand has occurred to color unique products such as toys, crayons, textile printing, and hand-made paper [80] using natural colorants, and biological pigments produced by microorganisms and plant source. These are being heavily investigated as potential sources to replace chemically synthesized colorants [79].

Biological pigments have antioxidant, antimicrobial and anticancer properties that make them relevant for use in various industries such as food, cosmetic and pharmaceutical industries [79]. The great diversity of biological pigments (anthocyanins, carotenoids, betalains, chlorophyll, and others) allows their use in a wide range of applications, resulting in a positive impact on the environment and human health [81].

There is massive interest in bioactive pigments that have beneficial health effects on humans, and consumers are driving the demand for natural food compounds such as bioactive pigments. Continuous exploitation of terrestrial or aquatic natural resources for natural food compounds will impose a huge demand on these resources. Hence, there is an ever-growing need for sustainable production of bioactive pigments to ensure continuity of use for future generations and allow a feasible increase in the utilization of these bioactive pigments [81]. Herein, a review of innovative and sustainable extraction methods of bioactive pigments is shown, highlighting the potential for mass production of bioactive pigments with diverse properties.

### 5.1. Bioactive Pigments from Food Wastes

Food waste is very important for the sustainability and economic development of food systems, since its utilization, management and valorization can benefit human health. Because of their bioactive compounds and bioactive pigments, food wastes have attracted the attention of research and industries in the last few decades [82]. Bioactive pigments are usually added to foods as additives to improve their appearance, since color is one of the major attributes that affect the consumer’s perception of food quality [76]. In this way, food colorants are one of the prime additives used in food products to enhance their appeal or give them a specific color [16,76].

**Figure 1:**
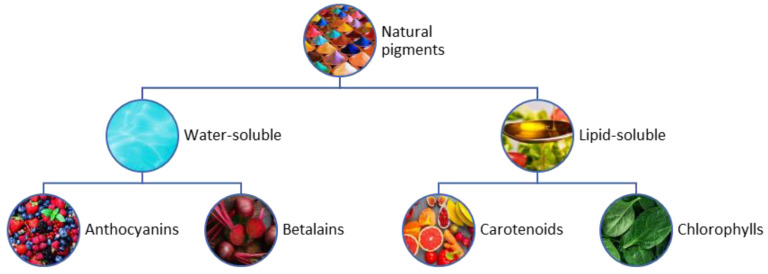
Classification of natural pigments (adapted from [83]).

In Figure 1, the classification of different natural pigments obtained from vegetal wastes is shown [83].

Anthocyanins (cyanidin, malvidin, delphinidin, peonidin, petunidin, and pelargonidin) are water-soluble pigments, and part of the phenolic family, contributing to various colors (red, orange, blue, and purple) in flowers, fruits, and vegetables [77,78]. Due to their antimicrobial and antioxidant properties, and the ability to change in color with the change in pH, they are considered as suitable for active packaging. Roselle anthocyanins have been reported as suitable raw materials in combination with biodegradable polymers for monitoring the freshness of fish and meat products [84].

Betalains (betacyanin and betaxanthin) are water-soluble phenolic pigments obtained from plants such as red pitaya, amaranth, beetroot, and prickly pear [77,78]. They have antimicrobial, antioxidant, anti-lipidemic, anti-diabetic, and anti-cancer properties. Like anthocyanins, betalains can be used as suitable substances for the development of active and intelligent packaging [84]. Although they are not reported as bioactive substances present in the main fruit crops of the world (the main focus of this review), they represent an interesting pigment derived from food waste, and mainly vegetable waste [85].

Carotenoids (lycopene, lutein, zeaxanthin, β-carotene, β-cryptoxanthin, and α-carotene) are lipid-soluble pigments mostly present in citrus wastes and vegetables (tomato, carrot, and sweet potato) [77,78]. These are among the most desirable bioactive pigments due to their color range (red, yellow, and orange) [83].

Chlorophylls (chlorophylls-a and chlorophylls-b) are lipid-soluble pigments that can be found in plants, cyanobacteria, and mosses [77,78]. Like the aforementioned bioactive pigments, chlorophylls possess antioxidant properties and have high nutrient value. Such properties prevent the oxidative damage of cells by eliminating free radicals and help to dispose of carcinogens by binding them in the gastrointestinal tract, and resisting cancer cell growth [84]. It is reported that chlorophyll acts as a time temperature indicator in the temperature range of 50 °C to 70 °C. For instance, an Amaranthus leaf extract in gelatin/polyvinyl alcohol (PVA)-based films can monitor the quality of chicken and fish effectively, as it is sensitive to volatile amines. Green tea and basil extracts consist of chlorophyll, which imparts a yellow-green color and undergoes a change in color at different pH levels [84].

Water-soluble pigments are used as colorants in consumer drinks, as reported by Sampaio et al. [86]. However, since these pigments are water soluble, their potential applications in the textile industry are limited and unknown. Nevertheless, natural pigments such as carotenoids and chlorophylls are potentially usable as natural bioactive compounds in the textile industry.

As previously pointed out, the extraction method is key to the sustainable production of bioactive compounds or bioactive pigments. In this regard, several studies have been developed to take full advantage of various food wastes (peels, leaves, and seeds) that are considered environmental hazards.

Table 9 presents multiple studies found in the literature in a summarized manner. In this way, readers of this review article can better understand and compare different extraction techniques and their results.

From the studies presented in Table 9, it can be concluded that several extraction methods (conventional and non-conventional) have been conducted in the extraction of valuable food wastes. From the studies found, some distinguish themselves by employing non-organic solvents and non-conventional methods.

Murador et al. [87], studied the use of ILs in the extraction of carotenoids from orange peel. In this study, four different ILs were tested: 1-butyl-3- methylimidazolium chloride ([BMIM][Cl]), 1-n-butyl-3-methylimidazolium hexafluorophosphate ([BMIM] [PF6]), 1-n-butyl-3-methylimidazolium tetrafluoroborate ([BMIM][BF4]), and 1-hexyl-3-methylimidazolium chloride ([HMIM][Cl]). RSM was applied to optimize the carotenoid extraction conditions. Results showed that [BMIM][Cl] was the most effective IL, surpassing the results obtained by acetone extraction.

Chedea et al. [90], also analyzed the carotenoid content saponified extracts from the peel of two orange varieties (Valencia and Navel). The data revealed important differences of carotenoid composition depending on orange variety since the Valencia variety was richer in short chain apocarotenoids, with the Navel variety richer in nonpolar carotenoids.

Murador et al. [91] studied xanthophylls, the main carotenoid class found in citrus fruit, that can be present in their free form or esterified with fatty acids, forming esters. The main aim of the study was to evaluate the carotenoid content of orange peel using alternative green approaches: extraction with ILs and SFE. Results showed that with ILs, the total carotenoid content was lower than with acetone, but it was still an improvement when compared to the previous study done by the author [87].

### 5.2. Bioactive Pigments Derived from Microalgae

Countries with vast coast lines have direct access to a very large and exclusive economic zone related to the marine environment. From this marine environment, immense potential can come if a blue bioresource-based economy is adopted that can meet current global economic, environmental, and societal demands [71]. Bioactive pigments such as astaxanthin (carotenoid) could play an important role in meeting society’s needs in the future.

Currently, less than 1% of the astaxanthin on the market comes from natural sources. Despite its benefits, around 99% of astaxanthin is still synthesized by big companies such as BASF and Hoffman-La Roche [71]. Panis and Carreon [92], proposed a model to perform a technical and economic analysis of the production of natural astaxanthin from *Haematococcus pluvialis* in two European cities (Livadeia and Amsterdam). From this study, it was concluded that naturally derived astaxanthin still cannot compete with the synthetic form, as the calculated production costs for the natural pigment were around EUR 1536/kg (in Livadeia) and EUR 6403/kg (in Amsterdam), while the synthetic production cost was EUR 880/kg [92]. Hence, there are still optimization studies missing on the extraction of natural pigments from microalgae to compete with synthetic options. In addition, the extraction processes must follow green chemistry principles in order to reach a more sustainable standard.

In a recent study Wils et al. [93], reported the first screening of NADES for the extraction of pigments and free fatty acids from *Spirulina platensis*. In their research, Wils et al. [93] evaluated NADES with a wide range of polarities, and the resulting extracts were compared according to their pigment and free fatty acid contents. After intensification of the extraction process, spirulina–NADES formulations and NADES alone were evaluated in terms of the effects on cutaneous inflammation induced by *Staphylococcus aureus*, as well as on four bacterial species that are part of the skin microbiota. The glycerol/glucose formulation exhibited an anti-inflammatory effect on keratinocytes stimulated by *S. aureus*. Nonpolar NADES alone and formulations impacted bacterial viability, especially for *S. aureus*, thus providing a new approach for the regulation of skin microbiota or product preservation.

In Table 10, a summary of different extraction studies of bioactive pigments from microalgae found in the literature is shown.

As shown in Table 10, several pigments (mainly carotenoids, chlorophylls, and phycocyanin) can be extracted from different species of microalgae. Although the reported yields are low, the studies report the use of biobased solvents such as NADES in the extraction process. Hence, despite the lower yields obtained, the principles of these studies are in line with green chemistry principles, and more research should be done towards the optimization of these extraction processes.

## 6. Market Analysis

It is evident that food wastes and microalgae are an extraordinary source of bioactive compounds and pigments. Due to the goals set by the United Nations [2], these compounds will gain more and more attention from the global research community, and efforts will be made towards the optimization of extraction processes and the formulation of different natural products derived from the two major sources of bioactive compounds that have been reviewed in this article.

Food wastes are a proven source of bioactive pigments and compounds with pharmaceutical and cosmetic applications, and are also used in biofuel production [96].

To take full advantage of the rich properties and compounds present in food wastes, it is necessary to understand the market potential of the food waste-derived products. For instance, in 2018, the global demand for pigments was approximately 9.7 million tons [79], and in 2014, 45% of the total demand was linked to the production of paints, the food sector, and the pharmaceutical sector. This demand may also trend upwards due to the growing market for the natural food colorants [79]. It is estimated that the universal food colorant market could reach 3.75 billion USD in 2022. One of the major contributors to this market is carotenoids. The global market polls estimated the market potential of carotenoids for the years 2016–2024 in foods, beverages, pharmaceuticals, cosmetics, animal feed and dietary supplements as 26.1, 9.2, 6.5, 34.8, and 23.5%, respectively [97]. Although the market of natural carotenoids is lower (24%) than synthetic carotenoids (76%) because of their high cost [64,91], natural carotenoids have many medicinal and health-improving properties that make them a key ingredient in nutraceuticals, make-up, and pharmaceuticals. Thus, carotenoids derived from food wastes are considered profitable business prospects for the food and healthcare sectors.

Food waste can also be converted into a spectrum of bio-commodity chemicals and bioenergy by employing acidogenic fermentation [96]. Bioconversion of food wastes into sustainable chemicals offers new alternatives for fossil-based chemicals. Food waste can be employed as a substrate in anaerobic fermentation for biogas production. In addition, research efforts have been extended towards the production of liquid biofuels, commodity chemicals, biohydrogen, and bioelectricity [96]. Such was the case studied by Ezekoye et al. [98], where 76.93% of citrus sinensis seed oil was successfully converted into biodiesel.

Microalgae are also a proven source of carotenoids [93]. Carotenoids from *Haematococcus* sp. and *Chlorella* sp. are sold at 40–50 USD per kg in the open market [99]. Microalgae have also been commercialized as a food supplement due to their lipid, carbohydrate, and protein content. Microalgal lipids are used in the food and pharmaceutical sector mainly because they contain the essential fatty acids eicosapentaenoic acid (EPA) and docosahexaenoic acid (DHA), which are absent from the main food crops, and some other high-value fatty acids (e.g., omega-3, γ-linolenic acid, etc.). On the other hand, lipids can be the feedstock for biodiesel production, though not all lipids are suitable, only the neutral lipids (including triacylglycerides (TAGs)). In the presence of a catalyst and alcohol, the lipids are converted to biodiesel (fatty acid methyl esters, FAME) [99].

### 6.1. Packaging Applications

The packaging of foods includes advanced technological methods mostly employed in developed societies. Protecting food from the harmful effects of oxygen scavengers, water vapor emitters, ultraviolet radiation, and contamination from microorganisms, and chemical agents constitutes, the main purpose behind food packaging [100]. In the food supply chain, fresh fruit packaging is vital from the farm until the product is received by the final consumer. Active food packaging functions by releasing active agents into the food that facilitate improved food quality with stability. The active ingredients within the food packaging system play an active role in the quality of food and durability either by acting as scavengers or inactivating deleterious compounds through the release of desirable components that have antimicrobial or antioxidant properties [100]. This new kind of packaging has emerged through changing patterns with respect to customer preferences regarding food products with a longer shelf life.

The use of biodegradable packaging systems reinforced by exploiting natural compounds is the latest trend to attract consumer demand in substituting synthetic preservatives in foods that can protect against food spoilage. Naturally derived biopolymers such as proteins, lipids, and polysaccharides, often serve as the base material for biodegradable packaging. They are recyclable, decompose in a short while, non-toxic, and are eco-friendly [84].

Many compounds such as anthocyanins, curcumin, betalains, tannins, chlorophyll, brazilin, and other phenolic compounds can be extracted from food waste. These compounds can act as indicators when mixed with a biodegradable polymer matrix, and provide significant results [84]. In addition, as shown by Kuntzler et al. [101], phenolic compounds found in microalgal extracts can also be effective against bacteria commonly found in human diets. Thus, application of phenolic compounds derived from microalgae could also play a big role in the packaging sector. Moreover, in a recent review by Karaduman et al. [102], multiple studies were summarized concerning the value of several microalgae-derived compounds in food packaging.

In Table 11, a summary of different packaging applications of bioactive compounds and pigments derived from food wastes and microalgae is presented.

### 6.2. Pharmaceutical Applications

As previously stated, food waste and microalgae-derived bioactive compounds and pigments are already in use in the pharmaceutical sector [36,40,57,66]. For example, di-hydrochalcones present in apple pomace can be used in the treatment of type-2 diabetes, obesity, and hyperglycemia attenuation [40], suggesting that these phenolic compounds may be useful in the regulation of sebum production, alleviating skin diseases such as acne (in which fat production is altered). Mangiferin (one of the predominant polyphenols in mango leaves) has shown multiple pharmaceutical properties such as antioxidant, antibacterial, antifungal, antidiabetic, immunomodulatory, anti-inflammatory, and analgesic properties, with potential uses in the treatment or prevention of chronic diseases including cancer and neurodegenerative and cardiovascular diseases [65], while one of the most well-known nuts worldwide (almond) stands out for its high level of α-eleostearic acid, which is effective in the suppression of the growth of cancer cells and possesses antihypertensive properties due to the presence of vasodilator compounds, such as ursolic acid and uvol [60].

Cherries have also been studied for their properties. For instance, extracts obtained from sweet cherry seeds are used in the food, pharmaceutical or cosmetic industries [25]. Additionally, as shown by Gonçalves [50], sweet cherry extracts have great biological potential, mainly due to their antioxidant activity against free radical species, and protecting cells against oxidative damages, and may even be used as a therapeutic in the treatment of inflammatory diseases (such as diabetes, gout, and arthritis), hemolytic anemia, cancer, neurological and cardiovascular pathologies [50].

Microalgae have also shown good pharmaceutical properties, since fatty acids present in oily microalgae extracts have antimicrobial, antiviral, antibacterial and antifungal properties, and have been widely used as ingredients for different skin care, sun protection, and hair care formulations [73].

In Table 12, a summary of pharmaceutical applications of bioactive compounds and pigments from microalgae and food wastes is presented.

### 6.3. Textile Applications

Textiles play an essential role in human life [104]. The value of the global fashion industry is US$ 3000 billion, which represents more than 2% of the gross domestic product (GDP) [105]. In the past two decades, the textile industry has doubled its production to meet the increase in global consumption from 7 to 13 kg per person. With higher rates of consumption, more and more clothes (around two thirds) are being disposed of in landfills at the end of use, and only around 15% are being recycled [105]. To significantly reduce the environmental and social impacts of the textile industry, radical changes are required, especially in the way in which textiles and clothes are designed, produced, traded, used, and recirculated [105].

To offset textile impacts on the environment, waste from food industries could be used in the manufacturing of textiles, thus reducing the environmental impact of these wastes and facilitating the recycling of clothes due to the use of bio-based textiles. Food wastes could be considered as primary or secondary feedstocks for biopolymer production by extraction or fermentation with pre-treatment, or without pre-treatment by solid-state fermentation to obtain fermentable sugars [105]. In addition, food waste can be a solution for the dyeing of clothes. Since agriculture and food processing industries generate a large amount of organic waste that still contains many bioactive pigments, their sustainable use in the dyeing of textiles would diminish the environmental burden of food waste disposal while creating value and substituting synthetic coloring materials [106].

For instance, Verma et al. [107], investigated the effect of biopolymer dyeing with natural dye (onion skins) on the functional properties (antibacterial and UV protection) of cotton fabric. The reddish colored skin of onions was collected from hostel waste (one of the major food waste generators [3]) and it was found that chitosan-treated cotton fabric dyed with onion skin showed 97.20% and 98.03% reduction in the growth of *Escherichia coli* (*E. coli*) and *Staphylococcus aureus* (*S. aureus*) bacteria, respectively. Moreover, the treated, dyed cotton fabric had a higher ultra-violet protection factor (UPF) value when compared to alum-treated dyed cotton fabric [105]. Additionally, in another interesting study conducted by Sanchez-Sanchez et al. [63], extracts obtained from mango leaves were successfully impregnated into textile polyester using supercritical carbon dioxide. The results showed that the extracts also presented antioxidant, bacteriostatic, and bactericidal activities [63].

Microalgae-based dyes were reported in a study done by Moldovan et al. in 2017 [108]. In the study, phycoerythrin, a red microalgae-derived pigment, was successfully extracted from fresh algal biomass and used in a laboratory pigment-printing process with cotton samples. Results showed that a light pink color could be obtained when applying the algae-derived pigment in a printing process. In conclusion, the algal pigments showed a high printing capacity on cotton substrates. Additionally, although not yet scientifically reported, news has been released [109] concerning the use of a black pigment from algae in the dyeing of clothes (i.e., tee-shirts). Although the study done by Moldovan et al. [108] failed to report the antibacterial capacity of the cotton samples, the aforementioned cases showed antioxidant and antibacterial properties, thus making these textiles potential antimicrobial textiles.

Antimicrobial textiles are functionally active textiles, which may kill microorganisms or inhibit their growth. These antimicrobial textiles can be used in a wide variety of applications including air filters, health care, hygiene, medical, sportswear, storage, ventilation, and water purification systems. Public awareness of antimicrobial textiles and growth in commercial opportunities has increased during the past few years, not only related to their antimicrobial properties, but for their durability and new color patterns. These unique characteristics are important for the clothing industry since many commercial brands are now focusing on such types of materials [104]. While textiles are mainly seen as covers for the human body, and layers of protection against adverse weather conditions, the market and consumer needs are pushing the textile industry to be ever more innovative and efficient, which in turn leads to cutting edge R&D programs and the production of highly functional products. In this context, functional fabrics are one of the most advanced developments, as they represent materials with new properties and added value [63].

Electrospinning (electrostatic fiber spinning) could play an important role in the manufacturing of functional antimicrobial textiles. Electrospinning is a widely used method in the field of nanotechnology, which uses electric fields to produce micro and nano fibers [110,111]. The popularity of electrospinning increased during the end of the 20th century when many publications started to appear. Many applications for electrospun fibers exist, such as wound healing, tissue engineering, and textiles [112]. In addition, electrospun fibers are advantageous due to their flexibility and large surface area, making them suitable for medical and textile application, because of their superior waterproofing properties and breathability compared to thicker fibers [105].

Many studies [113,114] have reported the use of the electrospinning techniques with biomass-derived compounds, and their use in wound healing and tissue engineering. Kim et al. [113], reported a nanofibrous dressing composed of a PCL/alginate/Spirulina nanofiber by a coaxial electrospun coating to improve physical properties while preserving water content and bioactive release of the main material, while Kwak et al. [114], added *Phaeodactylum tricornutum* (*P. tricornutum*) into a gelatin dope solution to evaluate its effect in the electrospinning technique. The addition of *P. tricornutum* extracts increased the conductivity of the dope solution and consequently the fiber diameter was reduced. The obtained nanofibers exhibited anti-microbial activity against *E. coli* and multidrug-resistant *S. aureus*. In addition, the nanofibers showed no cytotoxicity.

Although there have been studies focused on the dying of clothes with pigments derived from biomass (i.e., food wastes and microalgae), the manufacturing of micro and nanofibers with incorporated bioactive compounds and pigments extracted from the biomass sources for clothing purposes, this is still an untapped research topic and should be developed further to take full advantage of all electrospinning applications. In addition, as previously mentioned, the clothing industry is a major player in society and could help bring new market value to food waste and microalgae-derived bioactive compounds and pigments.

## 7. Conclusions and Future Work

It is evident that food waste and microalgae are important sources of bioactive compounds and pigments for biorefineries. Compounds such as fatty acids, phenolic compounds, carotenoids, and others, have shown interesting applications in various fields such as textiles, packaging, pharmaceuticals and energy. This review centers its attention in the major fruit crops produced and consumed in the world, while highlighting the great economic value of bioactive compounds and pigments with antioxidant activities. In addition, countries around the world have vast coastlines which are great sources of antioxidant compounds due to the presence of microalgal species. Thus, microalgal and fruit crops may be looked upon as a great biomass reservoirs, and could play major roles in the development of third-generation biorefineries.

Research efforts that have been made towards optimizing conventional and non-conventional extraction methods used in the main fruit crops worldwide are still not sufficient for industrial application. Hence, future work must be done towards the development of more sustainable extraction methods, while focusing on the following important factors: (1) use of non-organic solvents (such as ILs, DES, and NADES); (2) optimization of method variables; (3) scale-up and economic evaluations, and (4) life cycle assessments. Furthermore, academia and industries should work together by establishing partnerships and creating more knowledge about existing processing problems, wastes, and by-products. By doing so, products derived from food wastes and microalgae will be more competitive in the global market, and it will become more evident that biorefineries not only contribute greatly to solve several issues such as residue and waste management, environmental hazards, and health problems, but also contribute greatly to the global economy.

## Figures and Tables

**Table 1:** Summary of keywords/search terms used in the Science Direct database and the results obtained.

Search Terms	Science Direct	Web of Science
“green extraction” AND “microalgae” AND “bioactive compounds”	245	118
“green extraction” AND “food wastes” AND “bioactive compounds”	240	442
“green extraction” AND “microalgae” AND “bioactive pigments”	7	21
“green extraction” AND “food wastes” AND “bioactive pigments”	7	342
“NADES“ AND “microalgae” AND “bioactive compounds”	39	0
“NADES“ AND “food wastes” AND “bioactive compounds”	49	0
Total	587	923

**Table 2:** Summary of the main advantages and disadvantages of conventional and non-conventional extraction methods (adapted from [17]).

Method		Advantages	Disadvantages
Conventional	Maceration	Simple method Can used for bulk and small extractions.	Often presents low extraction yield Time consuming Large quantities of solvent
	Soxhlet	Simple method No filtration of extract required	Large volumes of solvent Long extraction times
Non-conventional	UAE	Quicker extraction times Higher efficiency Low temperatures Lower energy costs Higher extraction yield	Energy loss may occur due to wastage of ultrasonic energy though ultrasonic bath
	MAE	Simple method Low volumes of solvent required Low extraction times needed	May require filtration after extraction High instrument cost
	SC	High extraction yield Environmentally friendly process No product degradation Faster process	Use of co-solvents Extraction depends on flow rate High cost of equipment High pressure

**Table 3:** Summary of studies found in literature on the extraction of bioactive compounds from apple wastes.

Waste	Extraction Method	Bioactive Compounds Detected	Ref.
Pomace	Maceration, 60 min, Water	Polyphenols: gallic acid, catechin, chlorogenic acid, and rutin	[26]
	UAE, 30 min, Water		
	MAE, 1.5 min, Water		
	PLE, 5 min		
	Conventional extraction with temperature, 20 min, 80 °C, Ethanol/Water (50:50)	Polyphenols: hydroxycinnamic acids, flavanols, and chalcone (phoretin and phloridzin)	[28]
	UAE, 5 min, 20 °C, Acetone/Water (70:30)	Polyphenols: flavanols, dihydrochalcones (phloridzin and phloretin-20-xyloglucoside), flavonols and cinnamic acids (chlorogenic and caffeic acids)	[29]
Seed	UAE, 15 min, 40 Hz, Hexane/Ethanol/Acetone	Tocopherols (Vitamin E)	[30]
	Maceration, 2 h, 25 °C, Acetone/Water (60:40)	Polyphenols: phloridzin, quercetin, and epicatechin	[31]
	UAE, 5 min, 35 °C, n-hexane	Tocopherols (Vitamin E)	[32]
Peel	Maceration, Acetone, 5 min	Phenolic compounds: gallic acid, catechin	[33]
	Turbo-extraction, 30 min, 40 °C, Ethanol	Phenolic compounds: phenolic acids	[34]

**Table 4:** Summary of different studies that have been conducted for the extraction of bioactive compounds from wastes of orange (*Citrus sinensis*).

Waste	Extraction Method	Bioactive Compounds Detected	Ref.
Peel	UAE and PEF, Ethanol, 15–180 min, 20–80 °C	Hesperidin, polyphenols and vitamin C	[46]
	UAE, 400 W, 30 min, 50% Ethanol	Vitamin C and phenolics (mainly hesperidin)	[47]
Seed	Soxhlet, 40–60 °C, 6 h, Petroleum ether	Tocopherols	[48]
	Maceration, Methanol	Limonoids and phenolic compounds: flavanones, phenolic acids	[49]

**Table 5:** Summary of different studies that have been conducted for the extraction of bioactive compounds from wastes of different cherry varieties.

Waste	Extraction Method	Bioactive Compounds Detected	Ref.
Seeds	Maceration, Ethanol/Water, 80 °C	Phenolic compounds, flavonoids and flavonols	[25]
	Soxhlet, 6 h, Diethyl ether	Tocochromanols and polyphenols	[54]
	UAE, 5 min, 35 °C, n-hexane	Tocochromanols (tocopherol and sitosterol)	[55]
Pomace	Maceration, 2 h, Ethanol/Water (80:20)	Phenolic compounds	[50]
	Maceration, 2 h, Ethanol/Water (50/50)	Polyphenols	[56]
	MAE, 90 s, 900 W, 60 °C	Phenolic compounds	[57]
	Maceration, 30 min, 50 °C, Methanol/water (80/20)		
	UAE, 5, 10, 15 min, 24 kHz, 400 W, Room temperature, Methanol/Water (80/20)		

**Table 6:** Summary of different extraction procedures applied to almond by-products (almond hull, almond kernel, almond shell, and almond skin) for recovery of bioactive compounds (adapted from [60]).

Waste	Extraction Method	Bioactive Compounds Detected
Hull	Maceration, Ethanol/Acetone, 24 h	Phenolic and flavonoid compounds
	UAE, 51.2% Ethanol, 40 kHz, 300 W, 13 min	Phenolic acids and catechin
Shell	Soxhlet	Phenolic compounds
	SFE, Petroleum ether, 40–60 °C, 90 min, 11 kPa	Lignin
Kernel	MAE, NaOH, 2450 MHz, 800 W, 23–67 °C, 3 min	Phenolic compounds (Lignans)
	SFE, butane, −0.09 MPa	Phenolic, phylosterol, tocopherol and tocotrienol compounds
Skin	UAE, Water, 20 kHz, 100 W, 20 min	Phenolic compounds, lipids, and proteins.
	MAE, 70% Ethanol, 2450 MHz, 100 W, 60 s	Flavonol rutinosides

**Table 7:** Summary of different studies that have been conducted for the extraction of bioactive compounds from wastes of different mango varieties.

Waste	Extraction Method	Bioactive Compounds Detected	Ref.
Leaves	Enhanced solvent extraction using a mixture of CO_2_/Methanol (50%) at 120 bar and 100 °C	Polyphenols	[63]
Kernel	Maceration, 1 h, Room temperature, Ethanol/Water	Polyphenols (gallic acid, caffeic acid, rutin and penta-O-galloyl-b-D-glucose)	[64]
Peel	80% Acetone	Polyphenols	[65]
	Alcoholic maceration and maceration with pectinase	Flavanols	[66]

**Table 8:** Summary of different studies that have been conducted for the extraction of bioactive compounds from different species of microalgae.

Microalgae.	Extraction Method	Bioactive Compounds Detected	Ref.
*Chlorella* sp.	Maceration, DES	Lipids	[16]
	UAE, IL	Lipids	[74]
*Spirulina platensis*	SFE, CO_2_ and Ethanol/Water, 50 min	Tocopherols and fatty acids	[75]
	MAE, Methanol/Ethyl acetate/Light petroleum, 400 W, 1 bar, 15 min	Tocopherols and fatty acids	
	SFE, CO_2_ and Ethanol, 75 min	Tocopherols	[76]

**Table 9:** Summary of different studies on for the extraction of bioactive pigments from food wastes.

Waste	Extraction Method	Reported Yield	Ref.
Apple peel	Solvent extraction, 80% acetone	Cyanidin: 169.7 g/100 g of dry peel	[33]
Apple seeds	UAE, Hexane/Acetone, 15 min, 40 Hz	β-carotene: 1.370–25.800 μg/g of dry weight Lycopene: 0.080–5.370 µg/g of dry weight	[30]
Orange peel	UAE, Ionic liquid extraction: [BMIM][Cl]	Carotenoid: 32.08 ± 2.05 μg/g of dry peel	[87]
Orange peel	UAE, Acetone	Carotenoid: 7.88 ± 0.59 μg/g of dry peel	[87]
Orange peel	Soxhlet, Ethanol, 79 °C, 40:1 liquid/solid ratio	Phenolic pigment: 57 g/g of dry peel	[88]
Orange peel	UAE	β-carotene: 0.63 mg/100 g of dry peel	[47]
Orange peel	Solvent extraction, Ethanol: hexane (4:3)	β-carotene: 4.99 mg/100 g of dry peel	[83]
Orange peel	UAE and PEF, Ethanol, 20–80 °C, 15–180 min	Carotenoids: 52.98 μg/g of dry peel	[46]
Orange seeds	Soxhlet, petroleum ether, 40–60 °C, 6 h	Carotenoids: 0.32 mg/kg of dry mass	[48]
Mango peel	Solvent extraction, 80% acetone	Anthocyanins:203 ± 5.03; 326 ± 3.05 μg/g of dry peel Carotenoids: 73.5 ± 0.53; 81.0 ± 0.42 μg/g of dry peel	[65]
Sweet Cherry Skin	UAE, 70% Ethanol, 40 kHz, 100 W, 40 °C	Carotenoids: 12.2 mg/g of dry skin	[89]
Cherrie seed	Soxhlet, 6 h, diethyl ether	Carotenoids: 0.56–1.61 mg/100 g of dry mass	[54]
Cherrie pomace	Stirring, Ethanol/Water (80:20), 2 h	Anthocyanins: 1076.97–2183.55 µg/g of dry weight	[50]
Red beetroot	Maceration, methanol/water (80/20)	Betalains: 0.47 mg betanin/100 g and 0.26 mg betanin/100 g	[85]
Red beetroot	UAE, 37 and 52 °C, 165 W, 25 kHz, 90 min	Betalains: 4.20 and 2.80 mg/g of betacyanins and betaxanthins	[85]

**Table 10:** Summary of different studies on the extraction of bioactive pigments from microalgae.

Microalgae	Extraction Method	Reported Yield	Ref.
*Chlorella* sp.	Maceration, Acetone	Chlorophyll-a: 6 mg/L	[94]
*Stichococcus* sp.	Maceration, Acetone	Chlorophyll-a: 7 mg/L	[94]
*Spirulina* sp.	UAE, NADES: Glycerol/glucose/water	Chlorophylls: 0.50 mg/g Carotenoids: 0.22 mg/g Phycocyanin: 3.96 mg/g	[93]
	MAE, Methanol/Ethyl acetate/Light petroleum, 400 W, 1 bar, 15 min	Carotenoids: 629 ± 0.13 µg/g	[75]
	SFE, CO_2_ and Ethanol/Water, 50 min	Carotenoids: 283 ± 0.10 µg/g	
*Scenedesmus* sp.	UAE, 70 min, 60 °C, 40 KHz, 300 W, NADES: Fen-Thy.	Carotenoid (Lutein): 4.4 mg/g	[95]

**Table 11:** Summary of different packaging applications of bioactive compounds and pigments from microalgae and food wastes.

Waste	Application	Ref.
Apple peel	Anthocyanins impregnation for pH-sensitive packaging to evaluate food freshness and quality	[84]
Microalgae	Phenolic compounds extracted from *Spirulina* sp. were incorporated in the production of nanofibers with antibacterial activity	[101]
Microalgae	Phycocyanin derived from *Spirulina* sp. as bioindicator in food packaging due to changing pH	[103]

**Table 12:** Summary of different pharmaceutical applications of bioactive compounds and pigments from microalgae and food wastes.

Waste	Application	Ref.
Apple pomace	Di-hydrochalcones used in treatment of type-2 diabetes, obesity, or hyperglycaemia attenuation	[40]
Apple pomace	Phenolic compounds in regulation of sebum production	[40]
Apple seeds	Tocopherols (Vitamin E) prevention of heart disease and prostate cancer	[30]
Mango leaves	Mangiferin in prevention of chronic diseases including cancer and neurodegenerative and cardiovascular diseases	[65]
Almond	α-eleostearic acid which is effective in the suppression of the growth of cancer cells	[60]
Cherry seeds	Phenolic compounds in various pharmaceutical applications	[25]
Cherry pomace	Inhibition of α-glucosidase, treatment of inflammatory diseases (as diabetes, gout, and arthritis), hemolytic anemia, cancer, neurological and cardiovascular pathologies	[50]
Microalgae	Carotenoids in photoprotection of the skin against UV light, prevention of liver fibrosis and cancer (colorectal)	[71]
